# Multi‐trait genomic selection can increase selection accuracy for deoxynivalenol accumulation resulting from fusarium head blight in wheat

**DOI:** 10.1002/tpg2.20188

**Published:** 2022-01-19

**Authors:** Rupesh Gaire, Marcio Pais de Arruda, Mohsen Mohammadi, Gina Brown‐Guedira, Frederic L. Kolb, Jessica Rutkoski

**Affiliations:** ^1^ Crop Sciences Univ. of Illinois at Urbana–Champaign 1102 S. Goodwin Avenue Urbana IL 61801 USA; ^2^ H.M. Clause, Inc. 9241 Mace Blvd. Davis CA 95618 USA; ^3^ USDA–ARS Plant Science Research & Crop and Soil Sciences North Carolina State University Williams Hall 4114A Raleigh NC 27695 USA; ^4^ Agronomy Dep. Purdue Univ. 915 W State St West Lafayette IN 47907 USA

## Abstract

Multi‐trait genomic prediction (MTGP) can improve selection accuracy for economically valuable ‘primary’ traits by incorporating data on correlated secondary traits. Resistance to *Fusarium* head blight (FHB), a fungal disease of wheat (*Triticum aestivum* L.) and barley (*Hordeum vulgare* L.), is evaluated using four genetically correlated traits: incidence (INC), severity (SEV), *Fusarium* damaged kernels (FDK), and deoxynivalenol content (DON). Both FDK and DON are primary traits; DON evaluation is expensive and usually requires several months for wheat breeders to get results from service laboratories performing the evaluations. We evaluated MTGP for DON using three soft red winter wheat breeding datasets: two diversity panels from the University of Illinois (IL) and Purdue University (PU) and a dataset consisting of 2019–2020 University of Illinois breeding cohorts. For DON, relative to single‐trait (ST) genomic prediction, MTGP including phenotypic data for secondary traits on both validation and training sets, resulted in 23.4 and 10.6% higher predictive abilities in IL and PU panels, respectively. The MTGP models were advantageous only when secondary traits were included in both training and validation sets. In addition, MTGP models were more accurate than ST models only when FDK was included, and once FDK was included in the model, adding additional traits hardly improved accuracy. Evaluation of MTGP models across testing cohorts indicated that MTGP could increase accuracy by more than twofold in the early stages. Overall, we show that MTGP can increase selection accuracy for resistance to DON accumulation in wheat provided FDK is evaluated on the selection candidates.

## Core Ideas

- Multi‐trait genomic prediction models can predict deoxynivalenol content more accurately.
- FDK was the secondary trait that contributed most to improving prediction accuracy for DON.
- MTGP was more than twice as accurate than STGP for DON in early stages of testing.

**Abbreviations**
BLUE: best linear unbiased estimate
BLUP: best linear unbiased predictor
CV: cross‐validation
DH: days to heading
DON: deoxynivalenol content
FDK: *Fusarium* damaged kernels
FHB: *Fusarium* head blight
GS: genomic selection
IL: University of Illinois
INC: incidence
ISK: incidence, severity, and kernel damage
MT: multi‐trait
MT‐GBLUP: multi‐trait genomic best linear unbiased predictor
MTGP: multi‐trait genomic prediction
SEV: severity
SNP: single‐nucleotide polymorphism
SRWW: soft red winter wheat
ST: single‐trait
ST‐GBLUP: single‐trait genomic best linear unbiased predictor.

## 1 INTRODUCTION

*Fusarium* head blight (FHB) is a devastating disease of wheat (*Triticum aestivum* L.) causing >50% yield loss in susceptible cultivars when conditions are favorable for disease development. In addition, FHB causes severe financial losses to farmers through quality deterioration and contamination with toxic trichothecenes such as deoxynivalenol (DON) that can render the grain unacceptable for human or even animal consumption because of the health risks associated with these compounds (Kang & Buchenauer, 1999). Using resistant cultivars coupled with cultural practices, such as crop rotation and fungicide application, can effectively control FHB. However, developing resistant cultivars has been painstaking because FHB resistance is a quantitative trait conferred by many quantitative trait loci (Venske et al., 2019); artificial conditions are required to generate symptom expression that is suitable for phenotyping, phenotypic expression of FHB resistance interacts with the environment thereby reducing the accuracy of selection (Miedaner et al., 2001); and phenotyping FHB resistance is laborious and expensive. In addition, while significant progress was made, the limits of marker‐assisted selection for FHB breeding appear to have been reached, necessitating the use of more capable technologies that can use whole‐genome information for selection (Gaire et al., 2021).

Genomic selection (GS), which uses genome‐wide markers to predict breeding values, has been shown to accurately predict FHB resistance traits (Arruda et al., 2015; Dong et al., 2018; Liu et al., 2019; Rutkoski et al., 2012) in U.S. winter wheat germplasm. While these studies focused on predicting individual FHB resistance traits, they primarily used single‐trait (ST) genomic prediction models, which fail to capitalize on information available from the genetic covariances among the traits. Simulation and empirical studies evaluating multi‐trait (MT) genomic prediction (MTGP) models have shown that using multiple correlated traits as response variables leads to higher predictive abilities than traditional ST models (Bhatta et al., 2020; Hayes et al., 2017; Jia & Jannik, 2012; Lado et al., 2018; Rutkoski et al., 2016; Sapkota et al., 2020). Similarly, several studies in wheat have shown that by using genetically correlated secondary traits along with primary traits in MTGP models, higher prediction accuracies can be obtained (Guo et al., 2020; Lado et al., 2018; Montesinos‐López et al., 2019; Rutkoski et al., 2016; Ward et al., 2019). Although MTGP models are well established, few studies have been performed to evaluate its effectiveness for improving FHB resistance (Schulthess et al., 2018; Steiner et al., 2019).

*Fusarium* head blight resistance is typically evaluated by phenotyping four traits: incidence (INC), severity (SEV), *Fusarium* damaged kernels (FDK), and deoxynivalenol content (DON). Genetic correlations between these traits are positive and moderately high (Gaire et al., 2021). Incidence is the percentage of spikes in a sample that shows any disease symptoms regardless of its spread within a spike. Severity is the percentage of spikelets within a spike that show disease symptoms. *Fusarium* damaged kernels is the percentage of kernels in the sample that are diseased. In addition to these resistance traits, agronomic traits, such as days to heading (DH), are known to passively influence disease development (Buerstmayr et al., 2020). Both FDK and DON are considered to be the primary traits because they have economic value, whereas SEV and INC are secondary traits that have been useful for selection for reduced FDK and DON (Sneller et al., 2012; Rutkoski et al., 2012). Higher FDK in kernels significantly reduces test weight as well as milling quality by lowering flour yield and baking quality (Wegulo et al., 2021). Similarly, the Food and Drug Administration has established guidelines of 1 mg L^−1^ DON for finished flour products. Because of the reduced marketability, the milling industry imposes strict price discounts that penalize low test weight, kernel damage, and DON content, leading to reduced income for farmers. Additionally, DON can impact malting and brewing processes, imposing challenges to the beer industry (Schwarz, 2017).

**Core Ideas**

- Multi‐trait genomic prediction models can predict deoxynivalenol content more accurately.
- FDK was the secondary trait that contributed most to improving prediction accuracy for DON.
- MTGP was more than twice as accurate than STGP for DON in early stages of testing.

In winter wheat breeding programs across the United States, phenotyping for DON can only be done on relatively small numbers of samples, and the data are often only available after planting decisions have been made. In regions where FHB is a significant problem, winter wheat is harvested in summer (June–July) and the next cycle is planted in fall (September–October) providing the narrow timeframe of 3–4 months or less for breeders to evaluate breeding lines for DON before making selection decisions. Breeders typically submit a limited number of samples to a service laboratory that evaluates DON, and data are generated over a period of several months. In the absence of DON data, wheat breeders usually rely on indices such as FHB index (estimated as INC × SEV/100) and incidence, severity, and kernel damage (ISK) index (estimated as 0.3INC + 0.3SEV + 0.4FDK) for indirect selection. However, because the genetic correlation between DON and these indices is moderate, indirect response to is expected to be lower than direct selection for DON.

Multi‐trait GS could improve selection for reduced DON at early stages of testing when DON data are absent. Selection candidates could be genotyped and evaluated for INC, SEV, and FDK and then this information could be used in a genomic prediction model containing all phenotypic data on FHB resistance traits from previous years. This approach could improve selection accuracy for FHB resistance and enable breeders to reduce the length of their breeding cycles. However, the combination of traits that should be phenotyped when using a MT GS approach remains unknown.

In this study, we evaluated the potential of MTGP for DON and determined how it could be efficiently used in a breeding program. To do so, two publicly available datasets from the University of Illinois (IL) and Purdue University (PU) soft red winter wheat (SRWW) breeding programs that evaluated FHB resistance were used. Our objectives in this study were to (a) compare predictive abilities of MT models with the ST models, (b) identify opportunities for resource optimization by finding optimal combination of traits or through partial phenotyping to predict DON, and (c) validate the performance of MT models in early generation testing cohorts.

## 2 MATERIALS AND METHODS

### 2.1 Plant materials

In this study, we evaluated the potential of MT genomic prediction models for predicting DON using DH, INC, SEV, and FDK as secondary traits using two different diversity panels of SRWW, one from the IL and one from PU. The IL panel consisted of 237 SRWW lines including 185 lines developed at the IL small‐grains breeding program. Similarly, the PU panel consisted of 367 SRWW breeding lines from the small grains breeding program at PU.

A third dataset from the breeding pipeline of the small‐grains breeding program at the IL was incorporated to show the effectiveness of MTGP models in real breeding scenarios. The dataset consisted of lines from advanced and preliminary stages of testing during the 2019 and 2020 growing seasons. Lines in the preliminary stage have been selected during the previous year based on their agronomic performance. Lines in the advanced stage have been selected during the previous year based on their agronomic performance and their FHB resistance. In 2019, there were 72 and 337 individuals in the advanced and preliminary stages. From the 2019 preliminary trials, 135 lines were selected for 2020 advanced trials. Altogether, in 2020, there were 162 and 315 individuals in advanced and preliminary stages.

### 2.2 Genotyping

Genotyping for IL and PU panels was described in detail by Arruda et al., 2015 and Gaire et al., 2021, respectively. The breeding lines in the third dataset were genotyped using the same approach. In brief, the three populations were genotyped using genotyping‐by‐sequencing following the protocol reported by Poland et al. (2012). The genotyping‐by‐sequencing libraries were sequenced using Illumina HiSeq2000 for the three populations. After sequencing, the raw reads were aligned to the contemporary reference genome and single‐nucleotide polymorphism (SNP) markers were called. The three SNP datasets were then filtered such that SNP with heterozygosity >10%, >20% missing data, and minor allele frequency <5% were removed. The missing markers were imputed using the ‘mean’ method described in the rrBLUP package (Endelman, 2011). The total number of SNPs in the IL and PU panels were ∼38,000 and ∼14,000, respectively. Realized relationship matrices for both panels were estimated using the method described by vanRaden (2008) and implemented in the A.mat function available in the rrBLUP package (Endelman, 2011).

### 2.3 Phenotyping

FHB resistance in the IL panel was scored during the summers of 2013, 2014, and 2015 in Urbana, IL, using a randomized complete block design with two blocks each year. Similarly, the PU panel was scored during the summers of 2018 and 2019 in West Lafayette, IN, using a randomized augmented design with six incomplete blocks consisting of four repeated checks in each block. In 2018, a single experimental set was evaluated such that experimental lines were not replicated, and checks were replicated in each of the six incomplete blocks and in 2019; two experimental sets were evaluated such that experimental lines were replicated twice and the checks were replicated across blocks. The third population, advanced and preliminary breeding lines, was evaluated in the 2019 and 2020 seasons. For all three experiments, nurseries were inoculated using *Fusarium graminearum*‐infested corn spawn as described by Gilbert and Woods (2006). The field was mist irrigated during flowering to maintain high humidity for disease development. Phenotypic data were collected for DH, INC, SEV, FDK, and DON. Days to heading was recorded when >50% of the plants in each plot showed heads completely emerged from the boot at Zadoks stage 59 (Zadoks et al., 1974). The INC and SEV data were recorded 21 d after anthesis. The FDK phenotype was assessed by comparing the harvested grain samples with known FDK standards (Arruda et al., 2015). The DON concentration was quantified using gas chromatography–mass spectrometry as described by Fuentes et al. (2005) at the Department of Plant Pathology at the University of Minnesota.

### 2.4 Statistical analysis

All the statistical analyses were performed using ASReml‐R Version 4.0 (Butler et al., 2018) in R statistical programming language (R Core Team, 2021). For the IL panel, best linear unbiased estimates (BLUE)s of each line for FHB resistance traits were estimated using the following model:

(1) $$\begin{array}{ccc} Y_{\mathrm{ijk}} & & =\mu +\mathrm{Lin}e_{i}+\mathrm{Yea}r_{j}+\mathrm{Block}{\left(\mathrm{Year}\right)}_{\mathrm{jk}}\\ & & +\;{\left(\mathrm{Line}\times \mathrm{Year}\right)}_{\mathrm{ij}}+e_{\mathrm{ijk}} \end{array}$$

Where *Y_ijkl_* is the observed phenotype, μ is the overall mean, Line*_i_* is the fixed effect of the *i*th line, Year*_j_* is the random effect of *j*th year, Block(Year)*_jk_* is the random effect of *k*th block within the *j*th year, (Line × Year)*_ij_* is the random effect of interaction between *i*th line and *j*th year, and *e_ijkl_* is the random error term.

For the PU panel, BLUEs were estimated using the following model:

(2) $$Y_{ijk}=\mu +\mathrm{Lin}e_{i}+\mathrm{Se}t_{j}+\mathrm{IncBlk}{\left(\mathrm{Set}\right)}_{jk}+e_{ijk}$$

Where *Y_ijk_* is the observed phenotype, μ is the overall mean, Line*_i_* is the fixed effect of the *i*th line, Set*_j_* is the random effect of *j*th experimental set (3 experimental sets), IncBlk(Set)*_jk_* is the random effect of *k*th incomplete block within the *j*th experimental set, and *e_ijk_* is the random error term.

To estimate the broad‐sense heritability (*H* ^2^) on an entry‐mean basis, variance components were extracted fitting Equations 1 and 2 with Line effects as random. Heritability was estimated as follows:

(3) $$H^{2}=\frac{\sigma_{g}^{2}}{\sigma_{g}^{2}+\left(\frac{\sigma_{g\times y}^{2}}{y}\right)+\frac{\sigma_{e}^{2}}{ey}}$$

where $\sigma_{g}^{2}$, $\sigma_{g\times y}^{2}$, $\sigma_{e}^{2}$ are genotypic, genotype × environment, and error variances, *y* is the number of years (*y* = 3 and 2 for IL and PU panel, respectively), *e* is the number of replications within a year (*e* = 2 and 1 for IL and PU panel, respectively). For the PU panel, $\sigma_{g\times y}^{2}$ was not estimable and thus was removed from the equation.

We evaluated the genetic correlations among the traits using genetic variance–covariance matrices estimated fitting a multivariate random mixed model as follows:

(4) Y=μ+Zg+e

where **Y** is a matrix of BLUEs of *n* × *t* dimension (*n* individuals and *t* traits), **μ** is the means vector of length *n × t*, *g* is a vector of random effects of genotypes for all traits with g∼N(0,Σ⊗I), and **e** is a vector of residuals with e∼N(0,R⊗I), where **Σ** and **R** are the unstructured variance–covariance matrices for the genetic and residual effects for each individual in all traits, respectively. The BLUEs were used as the response variable originated from the Equations 1 and 2 for estimating the genetic correlations for the IL and PU panels, respectively.

### 2.5 Genomic prediction models and cross‐validation

Genomic prediction models were evaluated using fivefold cross‐validation (CV) repeated 50 times. The genotypic and phenotypic data were divided into equal sets of five. Four of the sets were used as the training set, while the remaining set was used as a validation set. Genomic prediction models were trained using phenotypic and genotypic data from training sets, and best linear unbiased predictors (BLUPs) of DON for individuals in the validation set were predicted. The predictive ability of the MT model was estimated as the correlation coefficients between genomic BLUPs for DON and BLUEs extracted from Equations 1 and 2 for the IL and PU panels, respectively, in the prediction set.

#### 2.5.1 Single‐trait genomic prediction model

The predictive ability of the ST model was estimated as the correlation coefficient between the DON BLUEs extracted from Equations 1 and 2, and the genomic BLUPs for DON extracted from the following ST genomic BLUP (ST‐GBLUP) model:

(5) y=Xb+Zg+e

where **y** is the vector of phenotypic value for individuals; **X** is the incidence matrix for the fixed effects including the overall mean and the effect of year; **b** is the vector of fixed effects; **Z** is the incidence matrix for the genotype effects; **g** is the vector of additive genotype effects, referred to as breeding values, assumed to follow multivariate normal distribution such that **g** ∼*N*(0, **K** $\sigma_{g}^{2}$) where **K** is the realized kinship matrix and $\sigma_{g}^{2}$ is the additive genetic variance. Finally, **e** is a vector of residual errors assumed to be independent and identically distributed according to *N*(0,$\sigma_{e}^{2}$).

#### 2.5.2 Multi‐trait genomic prediction model

The predictive ability of the MT model was estimated as the correlation coefficient between the DON BLUEs extracted from Equations 1 and 2 and the BLUP of DON extracted from the following MT genomic BLUP (MT‐GBLUP) model:

(6) y=μ+Xb+Zg+e

where **y** is a vector of length *n × t*, (*n* individuals and *t* traits), **μ** is the means vector of length *n × t*, X is a block diagonal matrix with fixed effect design matrices per trait on the diagonal and for each trait the design matrix is the same, **b** is the matrix of year effects of *y* × *t* dimension (*y* years and *t* traits), **Z** is a block diagonal matrix with random effect design matrices per trait on the diagonal. The random effect design matrices are equal for each trait. **g** is a vector of predicted genetic values of the individuals for all traits with g∼N(0,Σ⊗K), and **e** is a vector of residuals with e∼N(0,R⊗I), where **K** is the realized additive relationship matrix among individuals estimated from the markers, and **Σ** and **R** are the unstructured variance–covariance matrices for the genetic and residual effects between traits, respectively.

#### 2.5.3 Cross‐validation schemes

**FIGURE 1:**
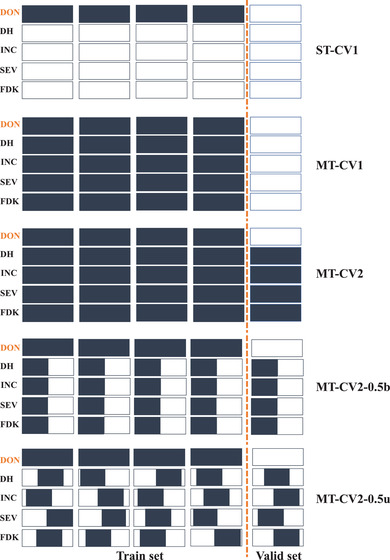
Five different schemes of cross‐validation (CV) modified from Lado et al. (2018). ST‐CV1, single fivefold CV where the training set had complete phenotypic data for deoxynivalenol (DON) and the validation set was not phenotyped for any trait. MT‐CV1, multi‐trait (MT) CV where phenotypic data on DON and the secondary *Fusarium* head blight (FHB) resistance traits were present in the training set and absent in the validation set. MT‐CV2, MT CV that uses all the genotypic and phenotypic information of individuals in the training set. MT‐CV2‐0.5b, MT CV where only 50% of the individuals in the training and validation sets had phenotypic data on all secondary traits in a balanced way. MT‐CV2‐0.5u, MT CV similar to MT‐CV2‐0.5b except the phenotypes were missing at random

Using 50 random sets of fivefold CV, we evaluated predictive abilities of ST‐GBLUP and MT‐GBLUP models in five different validation schemes that mimicked real breeding scenarios as described by Lado et al. (2018). In the second CV scheme, MT‐CV1, phenotypic data on DON and the secondary FHB resistance traits were present on the training set and absent in the validation set (Figure 1). In other words, MT‐CV1 did not use the phenotypic data of any of the traits from the validation set. Similarly, the third scheme, MT‐CV2, used all the genotypic and phenotypic information of individuals in the training set. It differs from MT‐CV1 in that it uses phenotypic data of secondary traits recorded on individuals in the validation set (Figure 1). In the fourth scheme, MT‐CV2‐0.5b, only 50% of the individuals in the training and validation sets had phenotypic data on all secondary traits (Figure 1). Similarly, for the fifth scheme, MT‐CV2‐0.5u, 50% of the secondary trait observations in the training and validation sets had phenotypic data on all secondary traits, but the phenotypic data were missing completely at random (Figure 1).

#### 2.5.4 Combination of traits

In this study, we considered DH, INC, SEV, and FDK as secondary traits and DON as the primary trait. We evaluated the predictive ability of the MT‐GBLUP model with all combinations of secondary traits for the prediction of DON. Altogether, 16 different combinations of models were evaluated including one ST model, four models with two response variables (bivariate), six models with three traits (trivariate), four models with four traits (quadrivariate), and one model with all the five traits (full model). Each combination of traits was evaluated using the same CV folds. In the small‐grains breeding program at the University of Illinois, the ISK index has been used to select for higher FHB resistance. The ISK index is calculated as (0.3INC) + (0.3SEV) + (0.4FDK) (Arruda et al., 2015). To compare our MT model's selection accuracies, we estimated the phenotypic correlation coefficients of ISK and FDK with DON in each cross‐fold for the best CV scheme.

#### 2.5.5 Validation in an applied breeding dataset

In the breeding dataset, there were four separate experiments: advanced 2019 and 2020 and preliminary 2019 and 2020. For each experiment and trait, BLUEs were extracted from the mixed model where trait was the response variable, the replicate effect was random, and the genotype effect was fixed. The BLUE estimates from 2019, including advanced and preliminary trials, were combined to use as training population. The ST‐GBLUP and MT‐GBLUP models, trained using 2019 data, were used to predict DON for lines tested in the 2020 advanced and preliminary trials. When predicting breeding values of lines in the 2020 advanced and preliminary trials, 135 lines in the 2020 advanced trial had phenotypic data for DON from when they were part of the 2019 preliminary trial, whereas for the 2020 preliminary trial, the phenotypic data for DON was completely missing.

## 3 RESULTS

### 3.1 Heritability and genetic correlation

In general, the broad‐sense heritability was higher for all the traits in the IL panel than in the PU panel. The heritability ranged from 0.58 for INC to 0.83 for DH in the IL panel, while for the PU panel, it ranged from 0.41 for INC to 0.73 for DH (Table 1). In both panels, genetic correlations between FDK and DON were much higher than the correlations between DON and INC and between DON and SEV. The correlation coefficients of INC and SEV with DON ranged from 0.20 to 0.25 in both panels. The genetic correlation between FDK and DON was 0.43 and 0.65 for the IL and PU panels, respectively (Table 1). The DH trait showed moderate positive correlation (*r* = 0.33) with DON in the IL panel, while there was no correlation between DH and DON in the PU panel (Table 1).

**TABLE 1:** Broad‐sense heritability (*H* ^2^) and genetic correlation (*r*_g_) of *Fusarium* head blight resistance traits with DON

**Panel**	**Trait**	***H* ^2^**	***r*_g_**
IL	DH	0.83	0.33
	INC	0.58	0.22
	SEV	0.78	0.23
	FDK	0.78	0.43
	DON	0.67	1
PU	DH	0.73	0.0
	INC	0.41	0.25
	SEV	0.45	0.20
	FDK	0.60	0.65
	DON	0.63	1

*Note*. DH, days to heading; INC, incidence; SEV, severity; FDK, *Fusarium* damaged kernels; DON, deoxynivalenol content.

### 3.2 Predictive abilities of ST vs. MT genomic prediction models

**FIGURE 2:**
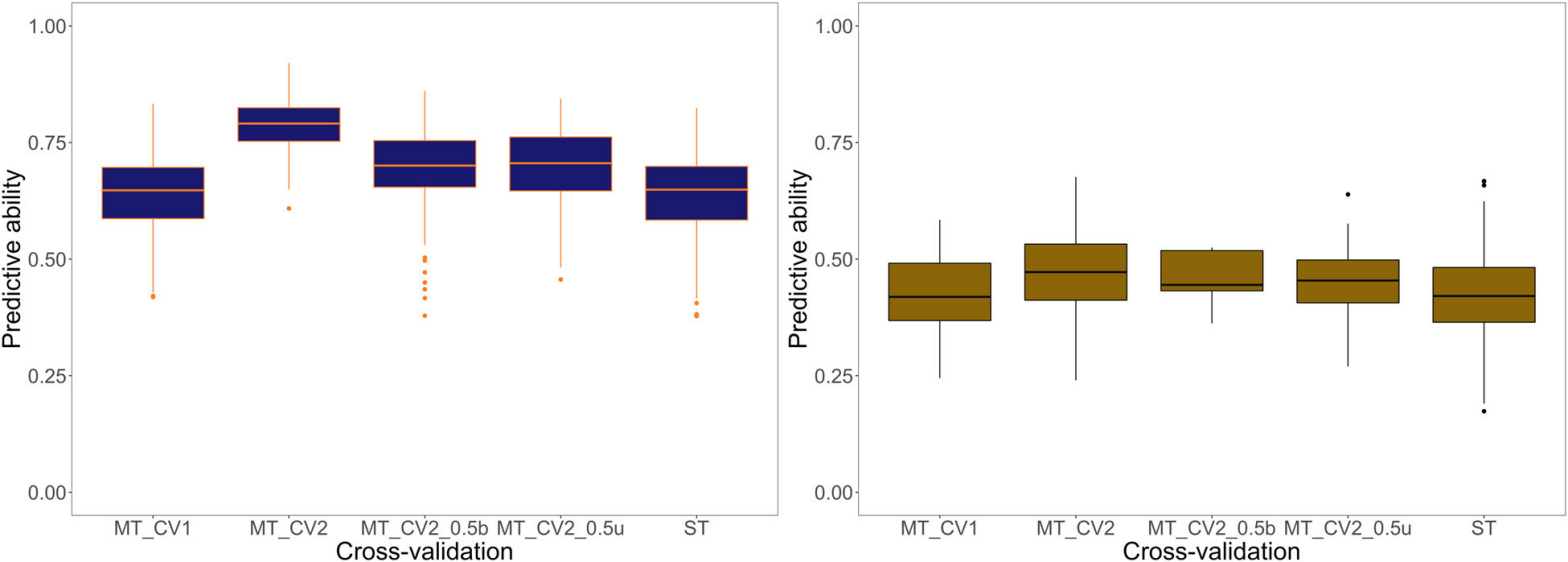
Predictive abilities for deoxynivalenol (DON) using single‐trait (ST), multi‐trait (MT) cross‐validation (CV) 1 (MT‐CV1), MT‐CV2, MT‐CV2‐0.5b, and MT‐CV2‐0.5u in the University of Illinois (IL) (left) and Purdue (right) soft red winter wheat populations. The MT genomic prediction models had all five traits: days to heading, incidence, severity, *Fusarium* damaged kernels, and DON content as response variables

We evaluated the predictive abilities of MT models using various CV scenarios that mimicked real breeding scenarios. MT‐CV1, the CV method that does not use secondary trait phenotypic data in the validation set, performed very similar to the ST model. The average predictive ability of both ST and MT models was 0.64 in the IL panel, whereas for the PU panel, the predictive abilities were 0.42 and 0.43, respectively (Figure 2). The other CV schemes (MT‐CV2, MT‐CV2_0.5b, and MT‐CV20.5u) showed higher predictive abilities than the ST model (Figure 2). For the IL panel, the predictive abilities of MT‐CV2, MT‐CV2_0.5b, and MT‐CV2_0.5u CV schemes were 0.79, 0.69, and 0.70, which were 23.4, 7.8, 9.4% higher than ST‐GBLUP, respectively (Figure 2), respectively. Similarly, for the PU panel, the predictive abilities of MT‐CV2, MT‐CV2_0.5b, and MT‐CV2_0.5u were 0.47, 0.46, and 0.45, which was 10.6, 8.7, and 7.1% higher than ST‐GBLUP, respectively (Figure 2).

### 3.3 Ability of MT models with different combinations of traits to predict DON

With DON as the trait of interest, we evaluated all possible combinations of traits in MT‐GBLUP to determine what trait combinations were most effective for improving predictive ability relative to ST‐BLUP. Because earlier analyses indicated that MT‐CV1 did not improve predictive ability relative to ST‐BLUP, this scenario was dropped from the analysis. To compare MT‐GBLUP with naïve indirect selection methods currently used by breeders, phenotypic correlation coefficients of ISK and FDK with DON were estimated for each fold in the fivefold CV.

**FIGURE 3:**
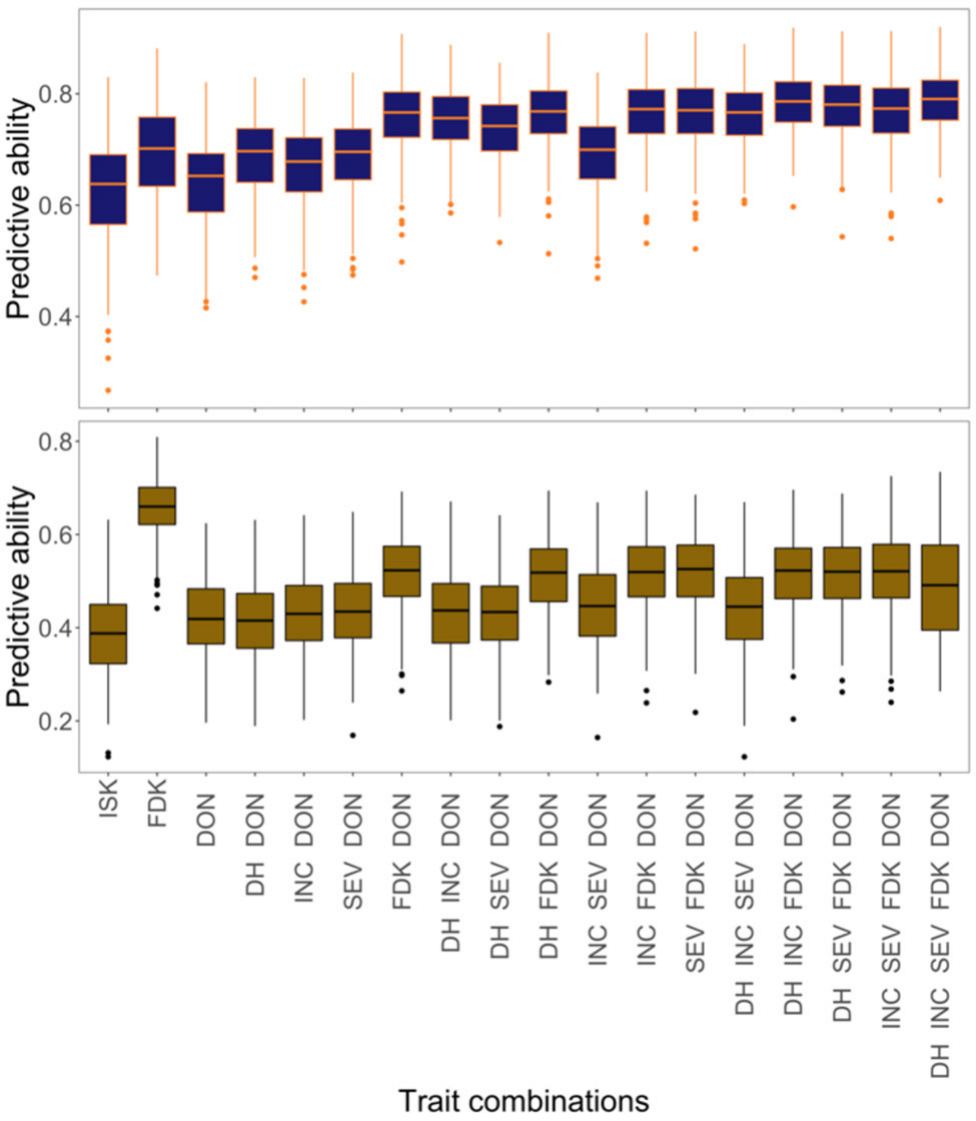
The predictive ability of incidence, severity and kernel damage (ISK) index, *Fusarium* damaged kernels (FDK), and the genomic prediction models to predict deoxynivalenol (DON) in the University of Illinois (IL) (top) and Purdue (bottom) soft red winter wheat populations. For ISK and FDK, the predictive ability was estimated as the phenotypic correlations with DON using data only from the validation set

In the IL panel with the MT‐CV2 scheme, bivariate models with DH, INC, or SEV had predictive abilities of 0.69, 0.67, and 0.69, respectively, which were only slightly higher than 0.64, the predictive ability of the ST model. (Table 2; Figure 3). Interestingly, the bivariate model with FDK in the response variable had a predictive ability of 0.76, which was comparable to that of the model that included all secondary traits. The trivariate and quadrivariate models had predictive abilities ranging from 0.74 to 0.78 except that the trivariate model with INC, SEV, and DON in the response variable had a predictive ability of 0.69 (Table 2; Figure 3).

**TABLE 2:** Mean predictive abilities of all combinations of traits as response variables in multi‐trait (MT) genomic prediction models across the four cross‐validation (CV) schemes

			**MT_CV1**		**MT‐CV2**		**MT_CV2‐0.5b**		**MT_CV2‐0.5u**	
**Panel**	**Trait combination** **^a^**	**Model type**	**IL**	**PU**	**IL**	**PU**	**IL**	**PU**	**IL**	**PU**
IL	DON	Univariate	0.64 ± 0.08^b^	0.42 ± 0.09	0.64 ± 0.08	0.42 ± 0.09	0.64 ± 0.09	0.42 ± 0.09	0.64 ± 0.09	0.42 ± 0.08
	DH DON	Bivariate	0.64 ± 0.08	0.42 ± 0.08	0.69 ± 0.07	0.41 ± 0.09	0.66 ± 0.08	0.42 ± 0.09	0.66 ± 0.08	0.42 ± 0.09
	INC DON	Bivariate	0.64 ± 0.08	0.42 ± 0.09	0.67 ± 0.07	0.43 ± 0.08	0.66 ± 0.08	0.42 ± 0.08	0.66 ± 0.08	0.43 ± 0.08
	SEV DON	Bivariate	0.64 ± 0.08	0.42 ± 0.08	0.69 ± 0.07	0.43 ± 0.08	0.66 ± 0.08	0.43 ± 0.09	0.66 ± 0.08	0.43 ± 0.08
	FDK DON	Bivariate	0.64 ± 0.08	0.42 ± 0.08	0.76 ± 0.07	0.52 ± 0.08	0.7 ± 0.08	0.47 ± 0.08	0.7 ± 0.08	0.47 ± 0.08
	DH INC DON	Trivariate	0.64 ± 0.08	0.42 ± 0.08	0.75 ± 0.05	0.43 ± 0.09	0.69 ± 0.08	0.42 ± 0.09	0.69 ± 0.08	0.43 ± 0.09
	DH SEV DON	Trivariate	0.64 ± 0.08	0.42 ± 0.08	0.74 ± 0.06	0.43 ± 0.09	0.7 ± 0.08	0.43 ± 0.09	0.7 ± 0.08	0.46 ± 0.08
	DH FDK DON	Trivariate	0.63 ± 0.08	0.42 ± 0.08	0.76 ± 0.06	0.51 ± 0.08	0.67 ± 0.08	0.46 ± 0.08	0.69 ± 0.08	0.45 ± 0.09
	INC SEV DON	Trivariate	0.64 ± 0.08	0.42 ± 0.08	0.69 ± 0.07	0.45 ± 0.09	0.7 ± 0.08	0.44 ± 0.08	0.7 ± 0.08	0.47 ± 0.08
	INC FDK DON	Trivariate	0.64 ± 0.08	0.42 ± 0.08	0.76 ± 0.07	0.52 ± 0.08	0.7 ± 0.08	0.46 ± 0.08	0.71 ± 0.08	0.46 ± 0.09
	SEV FDK DON	Trivariate	0.64 ± 0.08	0.42 ± 0.08	0.76 ± 0.07	0.52 ± 0.08	0.7 ± 0.08	0.46 ± 0.08	0.71 ± 0.08	0.46 ± 0.08
	DH INC SEV DON	Quadravariate	0.64 ± 0.08	0.42 ± 0.08	0.76 ± 0.05	0.44 ± 0.09	0.69 ± 0.08	0.45 ± 0.09	0.7 ± 0.08	0.46 ± 0.08
	DH INC FDK DON	Quadravariate	0.64 ± 0.08	0.42 ± 0.08	0.78 ± 0.05	0.52 ± 0.08	0.7 ± 0.07	0.44 ± 0.09	0.69 ± 0.08	0.43 ± 0.09
	DH SEV FDK DON	Quadravariate	0.64 ± 0.08	0.42 ± 0.08	0.78 ± 0.06	0.52 ± 0.08	0.71 ± 0.07	0.46 ± 0.07	0.7 ± 0.08	0.45 ± 0.09
	INC SEV FDK DON	Quadravariate	0.64 ± 0.08	0.42 ± 0.08	0.77 ± 0.06	0.52 ± 0.08	0.71 ± 0.07	0.45 ± 0.08	0.71 ± 0.07	0.46 ± 0.08
	DH INC SEV FDK DON	Pentavariate	0.64 ± 0.08	0.43 ± 0.08	0.79 ± 0.05	0.47 ± 0.09	0.69 ± 0.08	0.46 ± 0.07	0.7 ± 0.08	0.45 ± 0.08

*Note*. DH, days to heading; DON, deoxynivalenol content; FDK, *Fusarium* damaged kernels; IL, University of Illinois; INC, incidence; PU, Purdue University; SEV, severity.

^a^ The traits used as the response variables in the genomic best linear unbiased predictors model.

^b^ Mean and standard deviation of predictive ability represented as mean ± standard deviation.

Similar to MT‐CV2, the bivariate models in MT‐CV2_0.5b and MT‐CV2_0.5u schemes showed only a slight increase in predictive ability relative to ST‐BLUP (Table 2). The trivariate and quadrivariate models performed very similarly across both MT‐CV2_0.5b and MT‐CV2_0.5u schemes with the predictive ability ranging from 0.69 to 0.71 (Table 2).

In the PU panel with the MT‐CV2 scheme, bivariate models with DH, INC, or SEV had predictive abilities similar to that of the ST model (0.42). In contrast, the bivariate model with FDK had a predictive ability of 0.52, which is 24% higher than that of the ST model. Furthermore, all the trivariate and quadrivariate models that included FDK in the response variable showed similar predictive abilities to the bivariate model with DON and FDK as response variables (Figure 3). In contrast, the model without FDK as a response variable performed similar to the ST model. Interestingly, the full MT model with all the traits showed lower predictive ability than the trivariate and quadrivariate models with FDK in the response variable. For both panels, similar patterns of model performance were observed for the other CV schemes MT‐CV2_0.5b and MT‐CV2_0.5u. All the MT models with FDK as a response variable showed higher predictive abilities than the ST model and the bivariate models without FDK. Similarly, among the trivariate models, the model with FDK was slightly more predictive than the trivariate models without FDK (Table 2). The quadrivariate and full models showed similar prediction abilities ranging from 0.45 to 0.46 across both schemes.

The average correlation of FDK and ISK with DON within CV sets was exactly equal to actual phenotypic correlations. The phenotypic correlation of FDK and ISK with DON was 0.63 and 0.70, respectively, for the IL panel and 0.39 and 0.68 for the PU panel, respectively. In the IL panel, the phenotypic correlation of FDK and ISK with DON was lower than the predictive ability of trivariate, quadrivariate, and full models (Figure 3). Similarly, in the PU panel, the phenotypic correlation of ISK with DON was lower than the predictive ability of all the genomic prediction models. However, the phenotypic correlation of FDK (0.68) was much higher than the best MTGP model (0.47) (Figure 3).

### 3.4 Validation in breeding datasets

We evaluated the predictive ability of ST and a subset of MTGP models including bivariate model with FDK and DON; trivariate model with INC, FDK, and DON; the quadrivariate model with INC, SEV, FDK, and DON; and the full model to predict DON in the validation breeding dataset. Using genotypic and phenotypic data from 2019 advanced and preliminary trials as the training set to predict breeding values of lines in 2020 preliminary trials, the predictive abilities were 0.19, 0.35, 0.41, 0.30, and 0.30 for univariate, bivariate, trivariate, quadrivariate, and full models, respectively. Among the models evaluated, the MT model with INC, FDK, and DON in the response variable had the highest predictive ability, 115.8% more than the ST model. Similarly, using the same training set, the predictive abilities for DON among lines tested in the 2020 advanced trial were 0.38, 0.42, 0.43, 0.45, and 0.45, respectively. The quadrivariate model with DH, INC, FDK, and DON as response variables and the full model showed the highest predictive ability, 18.4% more than the ST model. The majority of the lines (83.3%) in the 2020 advanced trials were phenotyped for all five traits in the 2019 preliminary trials.

## 4 DISCUSSION

In this study, we evaluated the potential of MTGP models to predict DON. *Fusarium* head blight is a highly quantitative trait that has been dissected into several genetically correlated component traits that are most likely governed by a combination of independent and pleotropic genes. The MT‐GBLUP model should be advantageous when the phenotypic data consists of multiple correlated traits because it allows borrowing of information between traits as well as from relatives. Using publicly available datasets from two populations representing northern SRWW breeding programs, we evaluated the ability of MT‐GBLUP models to predict DON relative to ST‐BLUP models. Results from our CV experiments indicated that MT‐GBLUP can improve predictive ability for DON relative to ST‐GBLUP only when data on secondary traits are available within both training and validation sets. Similar results have been reported in cattle (*Bos taurus*), wheat, and barley (*Hordeum vulgare* L.) (Bhatta et al., 2020; Lado et al., 2018; Pszczola et al., 2013; Rutkoski et al., 2016; Ward et al., 2019). This demonstrates that gains in predictive ability in MT‐GBLUP primarily originates from information from correlated secondary traits in the validation set. Because secondary traits for DON can be evaluated at relatively early stages of testing, higher predictive abilities for DON using a multi‐trait genomic selection models including secondary traits on the selection candidates could enable shorter breeding cycles thereby increasing the rate of genetic gain for resistance to DON accumulation.

We observed clear patterns in the performance of MT‐GBLUP and ST‐GBLUP models within the IL and PU panels. Overall, predictive abilities and line‐mean heritabilities were higher with the IL panel than with the PU panel, which is expected given that heritability and GS accuracy are known to be inversely related (Combs & Bernardo, 2013; Goddard, 2009; Lorenzana & Bernardo, 2009). In addition, MT‐GBLUP outperformed ST‐GBLUP to a larger extent in the IL panel than in the PU panel, and this result is consistent with the observed trait correlations and heritabilities within the two panels. In simulation and empirical studies, prediction accuracies for low‐heritability traits are significantly higher when using MTGP models only when a correlated high‐heritability trait is available (Jia & Jannink, 2012; Rutkoski et al., 2016; Ward et al., 2019). In the IL panel, the heritability of DON is the lowest among all FHB traits, whereas in the PU panel, the heritability of DON is higher than all other traits. Thus, in the IL panel, the information gained from incorporating secondary traits in the GS model is greater and, therefore, has a larger impact on the predictive ability. In the PU panel, FDK was highly genetically correlated with DON, and both traits had similar levels of heritability. Thus, in the PU panel, models that included FDK data on the validation set showed higher predictive ability than those without FDK.

Although the results of this study clearly indicate that MT‐GBLUP outperforms ST‐GBLUP, MT‐GBLUP can only be recommended if it is more effective than indirect phenotypic selection methods that are already routinely used to select for lower DON. In this regard, mixed results were observed. In the PU panel, phenotypic correlations between FDK and DON were higher than the predictive abilities of all the genomic prediction models. This is surprising because the genomic prediction models use FDK data in addition to DON phenotypic information from relatives. In the IL panel, MTGP models showed higher predictive abilities than the phenotypic correlation with FDK, which is expected given that the genomic prediction models are using FDK data in addition to other data. It is possible that in the PU panel the MT‐GBLUP model performance may have suffered from inaccurate variance component estimation. The PU panel included phenotypic data from 2 yr, and within each year, an augmented design with four replicated checks was used. The low number of checks could have led to poor estimates of variance components, making MT‐GBLUP suboptimal and possibly less predictive of DON than FDK alone. With the IL panel, phenotypic data were collected across 3 yr with two replications within each year, thus variance component estimates were accurate and MT‐GBLUP optimal.

In the PU panel, the full model with all the five traits showed lower predictive ability, which might be due to zero genetic correlation of DH with DON. Jia and Jannink (2012) showed, through simulation, that for two traits without genetic correlation, MT‐GS was inferior to ST‐GS. The lower predictive ability might have occurred because of sampling of the population for five folds, which lead to nonzero estimates of correlation in the training population and then to erroneous information sharing across traits in the validation population.

In large wheat breeding programs, phenotyping for all the FHB resistance traits is expensive and time consuming. We evaluated the effects of partial phenotyping of secondary traits on predictive ability of MT models. Partial phenotyping for secondary traits improved predictive ability compared with the ST model; however, predictive abilities with partial phenotyping was lower than with complete phenotyping. Similar results were reported for milling quality in wheat and agronomic and malting quality in barley (Bhatta et al., 2020; Lado et al., 2018). Similarly, there was no significant difference in predictive ability from an unbalanced strategy (missing data completely at random) compared with the balanced strategy (missing data not at random) to reduce phenotyping. These results suggest that for resource management where phenotyping for all the traits is not possible, partial phenotyping for traits with high heritability and genetic correlations could be beneficial to improve the accuracy of selection (Jia & Jannink, 2012). Our results also suggest that many useful partial‐phenotyping approaches are possible.

The results from this study indicate that FDK is the most useful secondary trait for improving DON using MT GS. On the other hand, we found that INC and SEV alone did not improve the prediction accuracy of MT models. The FDK trait showed the highest genetic correlation with DON while having high heritability in both populations (Table 1). Thus, its influence on MT model predictive ability is expected (Jia & Jannink, 2012). Gaire et al (2021) found that FDK showed consistently higher genetic correlations with DON than with INC and SEV using data from 20 yr of FHB cooperative nurseries of northern SRWW breeding programs. This supports results of this study that FDK is invaluable for predicting DON in MT‐GBLUP. Since INC and SEV are less predictive of DON and are very laborious, expensive, and time sensitive, one could argue whether is it necessary to phenotype for both INC and SEV for predicting DON. Because this study found that that MT‐GBLUP models, with either INC and SEV, performed similarly, we suggest phenotyping for either INC or SEV to save resources without compromising selection accuracy for DON. Furthermore, if possible, identifying inexpensive and heritable novel traits that are genetically correlated to DON would be beneficial for improving prediction accuracy with limited resources. On the other hand, because we observed that the ISK index can be quite predictive of DON, breeding programs that are not implementing GS should continue to evaluate INC, SEV, and FDK to aid selection for reduced DON.

The results of this study evaluating MTGP models across testing cohorts showed that MT‐GBLUP was more than twice as accurate than the ST model. Ward et al. (2019) reported similar results in predicting yield in early generation trials using phenotypic data on the selection candidates. Increasing GS accuracy in a scenario where lines at later stages of testing are used to predict lines in earlier stage of testing is particularly useful for increasing the rate of genetic gain because this enables parent selection to occur sooner thereby reducing breeding cycle time. Overall, our results provide solid evidence that MT GS can increase the rate of genetic gain for DON improvement in wheat breeding programs.

## 5 CONCLUSIONS

*Fusarium* head blight s a devastating disease of wheat, and most of the SRWW breeding programs in the United States routinely evaluate breeding lines for FHB resistance. The phenotypic data for multiple correlated FHB resistance traits, including DH, INC, SEV, FDK, and DON, are routinely collected in these breeding programs. The MTGP models that exploit the information from genetic correlation among traits have potential to increase accuracy of prediction for FHB resistance. In this study, the potential of MTGP models to predict DON using different CV schemes was evaluated. Using phenotypic data for secondary traits evaluated on the selection candidates of interest substantially increased predictive ability relative to prediction based on marker data alone. Furthermore, it was observed that INC and SEV did not contribute much toward increasing the predictive ability of DON, while FDK improved predictive abilities substantially suggesting that phenotyping only FDK or FDK and either INC or SEV would save valuable resources for breeding programs without compromising the prediction accuracy of DON. To further improve MTGP for DON, identifying novel traits that are heritable and genetically correlated with DON would be a worthy objective.

## AUTHOR CONTRIBUTIONS

Rupesh Gaire: Conceptualization; Data curation; Formal analysis; Investigation; Methodology; Software; Visualization; Writing‐original draft; Writing‐review & editing. Marcio Pais de Arruda: Data curation; Investigation; Methodology; Resources; Validation; Writing‐review & editing. Mohsen Mohammadi: Data curation; Funding acquisition; Project administration; Supervision; Writing‐review & editing. Gina Brown‐Guedira: Data curation; Funding acquisition; Investigation; Methodology; Resources; Writing‐review & editing. Jessica Rutkoski: Conceptualization; Data curation; Formal analysis; Funding acquisition; Investigation; Methodology; Project administration; Resources; Software; Supervision; Validation; Visualization; Writing‐review & editing.

## CONFLICT OF INTEREST

The authors declare no conflict of interest.
